# Integrating proteomic, sociodemographic and clinical data to predict future depression diagnosis in subthreshold symptomatic individuals

**DOI:** 10.1038/s41398-019-0623-2

**Published:** 2019-11-07

**Authors:** Sung Yeon Sarah Han, Jason D. Cooper, Sureyya Ozcan, Nitin Rustogi, Brenda W.J.H. Penninx, Sabine Bahn

**Affiliations:** 1 Department of Chemical Engineering and Biotechnology, University of Cambridge, Cambridge, UK; 2 Department of Psychiatry, Amsterdam UMC, Vrije Universiteit, Amsterdam, The Netherlands; 3 Present Address: Owlstone Medical Ltd, 183 Cambridge Science Park, Cambridge, UK; 4 Present Address: Department of Chemistry, Middle East Technical University, Ankara, Turkey

**Keywords:** Diagnostic markers, Depression

## Abstract

Individuals with subthreshold depression have an increased risk of developing major depressive disorder (MDD). The aim of this study was to develop a prediction model to predict the probability of MDD onset in subthreshold individuals, based on their proteomic, sociodemographic and clinical data. To this end, we analysed 198 features (146 peptides representing 77 serum proteins (measured using MRM-MS), 22 sociodemographic factors and 30 clinical features) in 86 first-episode MDD patients (training set patient group), 37 subthreshold individuals who developed MDD within two or four years (extrapolation test set patient group), and 86 subthreshold individuals who did not develop MDD within four years (shared reference group). To ensure the development of a robust and reproducible model, we applied feature extraction and model averaging across a set of 100 models obtained from repeated application of group LASSO regression with ten-fold cross-validation on the training set. This resulted in a 12-feature prediction model consisting of six serum proteins (AACT, APOE, APOH, FETUA, HBA and PHLD), three sociodemographic factors (body mass index, childhood trauma and education level) and three depressive symptoms (sadness, fatigue and leaden paralysis). Importantly, the model demonstrated a fair performance in predicting future MDD diagnosis of subthreshold individuals in the extrapolation test set (AUC = 0.75), which involved going beyond the scope of the model. These findings suggest that it may be possible to detect disease indications in subthreshold individuals up to four years prior to diagnosis, which has important clinical implications regarding the identification and treatment of high-risk individuals.

## Introduction

Major depressive disorder (MDD) is a complex and burdensome disorder that is characterised by low mood and energy levels, as well as concentration problems, sleep disturbances and changes in weight and appetite^1,2^. It affects more than 300 million people worldwide^3^, and is estimated to become the most debilitating disorder worldwide by 2030^4^. Diagnosis of MDD currently relies on the evaluation of symptoms in clinical interviews according to the criteria outlined in the Diagnostic and Statistical Manual of Mental Disorders (DSM)-5^1^ or the International Statistical Classification of Diseases and Related Health Problems (ICD), 10th Revision^2^.

In recent years, the notion of subthreshold (or subsyndromal) depression has received increased attention^5–12^. The aim is to recognise those individuals experiencing depressive symptoms that do not fulfil the diagnostic criteria for MDD with respect to the number, severity and/or duration of symptoms (i.e., fewer than five diagnostic symptoms and/or duration of symptoms for less than two weeks based on the DSM-5), and as a result, are overlooked by the current checklist diagnostic approach. Although the operational definition of subthreshold depression varies across studies (as an officially agreed definition has not yet been established), they have collectively shown that the presence of subthreshold depressive symptoms is associated with increased functional and social impairment, reduced quality of life and increased utilisation of health services^8,10,13–15^. Importantly, while subthreshold depression has been identified as a risk factor for developing MDD in the future^13,16–18^, there is also growing evidence that targeting subthreshold individuals with indicated preventive interventions can help to prevent or delay the onset of MDD^19,20^. This highlights the clinical importance of finding early manifestations or biomarkers of incident MDD in subthreshold individuals, which could be used to identify those who will benefit most from appropriate preventive interventions. Early and more accurate detection of MDD is also essential for reducing the disease burden and the related healthcare costs.

The probability of an individual having or developing MDD can be predicted using a risk prediction model, based on his or her molecular, sociodemographic and/or clinical characteristics^21,22^. The clinical utility of such a model is in aiding the process of decision-making with regards to the diagnosis or treatment of patients or symptomatic help-seekers, and it is important that the performance of the model is reproduced when applied to new patient data. However, as the development of a prediction model in biomarker studies often involves performing model selection on high-dimensional data (small sample size relative to the number of features), model reproducibility can be limited by problems such as overfitting, which is when the model captures not only the underlying relationship of interest but also noise in the data, as well as model selection uncertainty, which is when no single model is strongly supported by the data. These problems need to be appropriately addressed to ensure that a robust and generalisable model is obtained.

A standard approach employed by many biomarker studies is to use healthy controls as a reference population against which patients are compared; however, in this study, we examined a more clinically relevant and appropriate sample population by defining individuals presenting with subthreshold levels of depressive symptoms. We developed a disease prediction model of MDD by comparing subthreshold individuals who did not develop MDD (reference group) against first-episode MDD patients, based on their proteomic, sociodemographic and clinical profiles. As the reference group was more similar to the patient group, this provided an additional challenge to model selection. We implemented several methods to limit model overfitting and ensure model generalisability, and in the presence of model selection uncertainty, applied feature extraction and model averaging across a set of candidate models to obtain an average prediction model. To investigate the prediction of future MDD onset, we then extrapolated this model to differentiate between subthreshold individuals who developed and did not develop MDD.

## Materials and methods

### Clinical samples

This study investigated participants from the Netherlands Study of Depression and Anxiety (NESDA)^23^, a naturalistic, longitudinal study in which 2981 participants (aged 18–65 years) were recruited from the general population and mental healthcare centres between 2004 and 2007 and followed-up for up to eight years. ﻿The protocol was approved by all relevant ethical committees (the Ethical Review Board of the VU ﻿University Medical Centre and by the local ethical review boards at the participating centres of the Leiden University Medical Centre and the Groningen University Medical Centre), and written informed consent was obtained from all participants^23^. Diagnoses of MDD and other psychiatric disorders were determined at the baseline and follow-up assessments using the Composite Interview Diagnostic Instrument (CIDI) for DSM-IV^24^.

For the purpose of this study, we selected 209 participants based on their disease status at the baseline and second- and fourth-year follow-up assessments, and baseline data of the 30-item Inventory of Depressive Symptomatology (IDS_30_; self-report)^25^, which measures the severity of depressive symptoms in the past seven days on a scale of zero (none) to three (severe). Using 16 items of the IDS_30_ corresponding to nine diagnostic symptoms that comprise the DSM-5 MDD criteria (Supplementary Table 1), we defined ‘subthreshold depression’ at baseline as presenting with two or more depressive symptoms, including at least one of sadness or anhedonia (two core symptoms of the DSM), whereby a symptom was considered as present if any one of the corresponding IDS_30_ items was above zero. To identify early biomarkers or indicators of MDD, we would ideally test for differences between subthreshold individuals who later developed and did not develop MDD; however, as the number of subthreshold individuals who developed MDD was limited, we first trained the model to differentiate between 86 subthreshold individuals who had no current or lifetime diagnosis of MDD at the baseline assessment and did not develop MDD by the fourth-year follow-up assessment (reference group) and 86 recent-onset MDD patients who experienced their first and only major depressive episode within a month before the baseline assessment (training set patient group; to provide a fair comparison, we ensured that they also fulfilled the criteria for baseline subthreshold depression). We subsequently extrapolated the model to predict the probability of developing MDD in the shared reference group and 37 subthreshold individuals who had no current or lifetime diagnosis of MDD at the baseline assessment and developed MDD by the second-year (*n* = 21) or fourth-year (*n* = 16) follow-up assessment (extrapolation test set patient group).

Sociodemographic information of the participants was collected at the NESDA baseline assessment^23^. This included sex, age, body mass index (BMI), education level, physical activity, smoking, alcohol abuse, recreational drug use, employment status, family history, childhood trauma, chronic diseases and medication use (Table 1). Clinical features were derived from the baseline IDS_30_ data: 28 depressive symptoms were derived from 30 IDS_30_ items (after items on increase or decrease in weight and appetite were aggregated into single domains of weight/appetite increase or decrease), and the IDS_30_ total score and severity classification were determined.

**Table 1:** Sociodemographic and health characteristics of individuals in the training set patient group (first-episode MDD patients), the extrapolation test set patient group (subthreshold symptomatic individuals who developed MDD within two or four years) and the shared reference group (subthreshold symptomatic individuals who did not develop MDD within four years)

	Shared reference group	Patient group	
		Training set	Extrapolation test set
*N*	86	86	37
Sex % (male/female)	35/65	48/52	32/68
Age (years)	37.8 (14.1)	41.8 (12.2)	38.5 (14)
Body mass index (kg/m^2^)	23.8 (4.4)	26.7 (6)	25.6 (5)
Education, % (basic/intermediate/high)	8/42/50	6/67/27	3/59/38
Physical activity, % (low/moderate/high)	23/48/29	30/44/26	27/41/32
Smoking, % (yes/no)	31/69	38/62	32/68
Alcohol abuse, % (yes/no)	21/79	40/60	19/81
Weekly alcohol consumption (number of drinks per week)	8 (11)	6.8 (11)	6.3 (7.4)
Recreational drug use (past month), % (yes/no)	7/93	8/92	8/92
Partner, % (yes/no)	69/31	57/43	70/30
Children, % (yes/no)	44/56	48/52	51/49
Employment, % (employed/unemployed/retired/occupationally disabled)	77/17/2/3	60/17/2/20	65/30/0/5
Absent from work due to health problems (past 6 months), % (yes/no/not applicable)	43/35/22	41/21/38	35/32/32
Childhood life event index score	0.3 (0.5)	0.2 (0.5)	0.2 (0.5)
Childhood trauma index score	0.5 (0.9)	1 (1.3)	0.8 (1.1)
Number of negative life events (past year)	1 (1)	1 (1.1)	0.9 (1)
Family history, % (yes/no)	73/27	87/13	84/16
Heart disease, % (yes/no)	1/99	5/95	5/95
Diabetes, % (yes/no)	3/97	6/94	11/89
Other chronic disease, % (yes/no)	26/74	40/60	22/78
Anti-inflammatory drug, % (yes/no)	2/98	8/92	8/92
Heart medication, % (yes/no)	10/90	23/77	8/92
IDS_30_ total score	14.9 (7.4)	37.4 (11.5)	20.2 (8.8)

*IDS* inventory of depressive symptomatology, *MDD* major depressive disorder

Numerical features are shown as the mean (standard deviation)

### Targeted protein quantification

Blood serum samples were collected at the NESDA baseline assessment, and prepared in a 96-well plate format using a liquid-handling robotic system, as described previously^26^. In this study, 77 proteins (146 peptides; Supplementary Table 2) were investigated using targeted multiple reaction monitoring (MRM) mass spectrometry (MS) analysis. The majority of these proteins was previously associated with psychiatric disorders^27^. Serum samples were diluted with ammonium bicarbonate, and dithiothreitol and iodoacetamide were used to perform disulphide bond reduction and cysteine alkylation, respectively. Proteins were digested overnight using trypsin (see Supplementary Information). Stable isotope-labelled internal standard (SIS) peptides were spiked for each endogenous peptide.

Trypsin-digested peptides were separated and detected using a liquid chromatography (LC) system coupled with a triple-quadrupole (QQQ) mass spectrometer (Agilent Infinity 1290 LC system and Agilent 6495 QQQ LC/MS system with Agilent Jet Stream electrospray ionisation (ESI) technology) (see Supplementary Information). Target proteins were represented by unique peptide sequences, and peptides were quantified at the transition level (three to four interference-free transitions were selected for each peptide, as described previously^26^). Peptide quantification was based on the peak area values of the endogenous and the SIS peptide transitions.

### Statistical analysis

#### Data pre-processing

Raw MS data were processed using Skyline software package (version 3.1.0)^28^. Statistical data pre-processing and analysis were carried out using R statistical software (version 3.4.4)^29^. A quantifier transition was selected for each peptide as the most abundant transition (highest peak area value) in both the endogenous and the SIS. Peptide quantification was based on the relative abundance of the endogenous and the SIS quantifier transitions, reported as the abundance ratio. The abundance ratio was log_2_-transformed for statistical analysis. No outlier samples were identified based on principal component analysis (PCA) of peptide abundance ratios (Supplementary Fig. 1).

There were no peptides with missing values, and no sociodemographic or clinical variables with more than 5% missing values. Missing values in sociodemographic and clinical variables were replaced using multiple imputation (R package mice^30^). Categorical variables were represented as sets of dummy variables. See supplementary Information for more details on data pre-processing.

#### Model selection

A total of 198 features (146 proteomic, 22 sociodemographic and 30 clinical) were analysed for model selection (Supplementary Table 3). Least absolute shrinkage and selection operator (LASSO) is a penalised regression method that reduces overfitting by performing shrinkage and model selection simultaneously^31^. Variables with poor discriminatory power are eliminated from the model as their coefficients are reduced to zero, whereas variables with non-zero coefficients are selected. We employed group LASSO regression^32^ (R package gglasso^33^) to allow sets of dummy variables derived from categorical variables to be selected together. To further reduce overfitting, we used ten-fold cross-validation to select the value of the shrinkage parameter lambda that resulted in the most regularised model. The data were randomly partitioned into ten folds, and each fold was retained as the test data, whilst the remaining nine folds were used as the training data for each round of cross-validation.

We generated 100 models by repeatedly applying group LASSO regression with ten-fold cross-validation on the training set. This allowed us to investigate model selection uncertainty by evaluating the sensitivity of model selection to small changes in the data that resulted from the random partitioning in ten-fold cross-validation. For each feature, we measured the proportion of models out of 100 in which it was selected, called the selection fraction. This was a value between 0 and 1 and used to assess the relative importance of the features. We identified unique models based on the combination of features selected and measured the frequency of occurrence of each unique model.

#### Akaike information criterion and Akaike weights

We implemented model averaging using the Akaike’s information criterion (AIC), as described in Burnham and Anderson^34,35^. The AIC measures how well a model approximates the given data (on a relative scale), where the model with the lowest AIC value is considered to be the best model^36^. We adopted the bias-corrected version of AIC (AIC_c_), as the sample size (*n*) was small compared with the largest value of *k*, where *k* is the number of features selected in a model (*n/k* ≤ 40^37,38^). We used this to compute the Akaike weight (*w*) of each model, interpreted as the probability that the model was the best approximating model for the data^34^. The weight was a value between 0 and 1, and the sum of weights of all models was equal to 1. For each unique model, we summed the weights of all corresponding models to estimate the probability that the selected combination of features comprised the best approximating model^27^. See Supplementary Information for the formulae used for the calculations of AIC_c_ and Akaike weight.

#### Feature extraction and model averaging

When there was one strongly supported unique model (e.g. *w* > 0.9), parameter estimation and prediction could be based on that model alone^34^. In this instance, we estimated the coefficients of the features in the dominant unique model by averaging over the corresponding set of models. However, in the absence of a dominant unique model, that is, when there was uncertainty in model selection, we implemented feature extraction and model averaging using all 100 models to obtain more reproducible predictions of the probability of MDD outcome. We included only features with selection fractions greater than or equal to 0.9 in the prediction model (feature extraction) to limit overfitting, and subsequently averaged over the 100 models (model averaging) to obtain better estimates of feature coefficients^34,39,40^. The weighted average coefficient of a given feature across a set of *R* models, $\widehat {\bar \beta }$, was computed:

$$\widehat {\bar \beta } = \mathop {\sum }\limits_{i = 1}^R w_i\hat \beta _i$$

where *w*_*i*_ and $\hat \beta _i$ were the Akaike weight and the estimated coefficient of a feature in model *i*, respectively. Models in which a feature was not selected contributed nothing to the average coefficient estimate, resulting in the shrinkage of the coefficient towards zero.

#### Predictive performance

We assessed the predictive performance of the models when applied to the training and extrapolated test sets by measuring the area under the receiver operating characteristic (ROC) curves (AUC) (R package ROCR^41^). The AUC is the probability that a randomly chosen individual with the disease is ranked higher than a randomly chosen individual without the disease (AUC: 0.9-1 = excellent; 0.8–0.9 = good; 0.7–0.8 = fair; 0.6–0.7 = poor; 0.5–0.6 = fail)^42^.

## Results

One hundred and forty-six proteomic, 22 sociodemographic and 30 clinical features (198 total) were measured in the training set patient group of 86 first-episode MDD patients, the extrapolation test set patient group of 37 subthreshold symptomatic individuals who developed MDD within two or four years, and the shared reference group of 86 subthreshold symptomatic individuals who did not develop MDD within four years (Table 1).

### Analysis 1: model selection including IDS_30_ total score

**Fig. 1:**
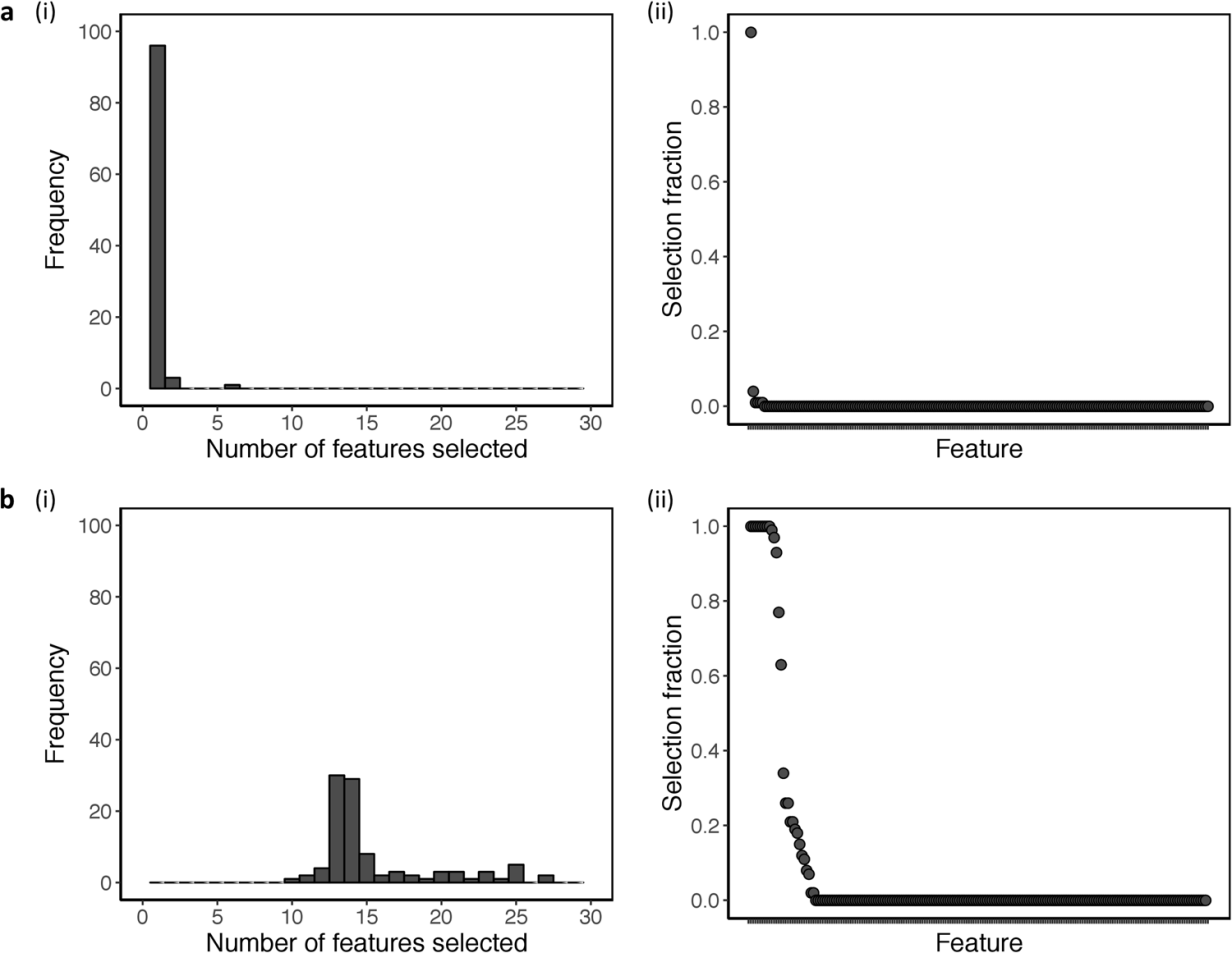
Feature selection across 100 models obtained from repeated application of group LASSO regression with tenfold cross-validation on the training set. **a** Analysis 1: model selection including IDS_30_ total score (198 features). **b** Analysis 2: model selection excluding IDS_30_ total score (197 features). (i) The number of features selected in each model. (ii) Selection fractions of each feature

When all 198 features were used, there was minimal uncertainty in model selection. The number of features selected in a model ranged from one to six with an average of one (Fig. 1a). IDS_30_ total score was selected 100 times, one peptide was selected four times, and four peptides were selected once; the remaining features were never selected (Supplementary Table 3). Three unique models were identified based on the combination of features selected (Supplementary Table 4). The most frequently occurring unique model consisting of the IDS_30_ total score alone occurred 96 times and had a model probability of 0.98. Given the strong support for this unique model (Model 1), there was no need for feature extraction, and the average feature coefficient was estimated using the corresponding 96 models (Table 2).

**Table 2:** Features included in the two prediction models

Feature	Model	Selection fraction	Average coefficient
*Proteomic*			
AACT_ADLSGITGAR	2	1.00	0.122
APOE_ALMDETMK	2	0.99	−0.195
APOH_EHSSLAFWK	2	1.00	0.08
FETUA_HTLNQIDEVK	2	0.97	0.082
HBA_MFLSFPTTK	2	1.00	0.231
PHLD_NQVVIAAGR	2	1.00	0.286
*Sociodemographic*			
BMI	2	1.00	0.291
Childhood trauma	2	1.00	0.115
Education; intermediate	2	0.93	0.065
Education; high	2	0.93	−0.055
*Clinical*			
Sadness; mild	2	1.00	−0.681
Sadness; moderate	2	1.00	0.819
Sadness; severe	2	1.00	0.369
Fatigue; mild	2	1.00	−0.124
Fatigue; moderate	2	1.00	0.339
Fatigue; severe	2	1.00	0.085
Leaden paralysis; mild	2	1.00	−0.145
Leaden paralysis; moderate	2	1.00	0.219
Leaden paralysis; severe	2	1.00	0.272
IDS_30_ total score	1	1.00	0.346

*IDS* inventory of depressive symptomatology, *BMI* body mass index, *AACT* alpha-1-antichymotrypsin, *APOE* apolipoprotein E, *APOH* apolipoprotein H, *FETUA* fetuin-A, *HBA* haemoglobin subunit alpha, *PHLD* glycoprotein phospholipase D

Model 1 (one feature) was based on the dominant unique model in Analysis 1 (model selection including IDS_30_ total score), and Model 2 (12 features) was developed by implementing feature extraction and model averaging in Analysis 2 (model selection excluding IDS_30_ total score) in the absence of a dominant unique model. The selection fraction and the average coefficient of the features are shown. Proteomic features are represented in a Protein_Peptide format. Categorical features (education, sadness, fatigue and leaden paralysis) are represented as sets of dummy variables

**Fig. 2:**
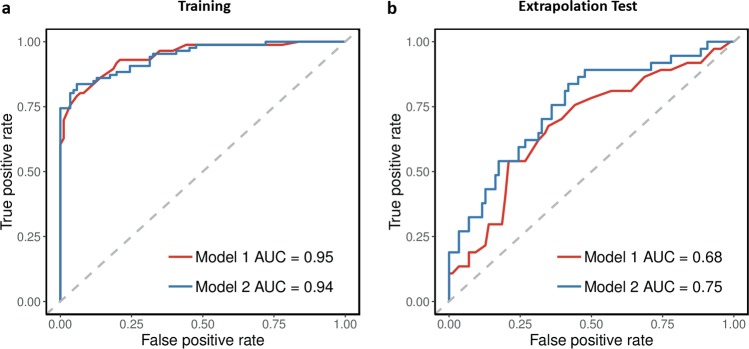
ROC curves showing model performance in predicting the probability of MDD outcome. Model 1 consisted of IDS_30_ total score and Model 2 consisted of six proteins, three sociodemographic factors and three symptoms. The prediction models were applied to predict the probability of MDD outcome in: **a** the training set (86 first-episode MDD patients vs 86 subthreshold individuals who did not develop MDD within four years), and **b** the extrapolation test set (37 subthreshold individuals who developed MDD within two or four years vs 86 subthreshold individuals who did not develop MDD within four years). AUC area under the curve, IDS inventory of depressive symptomatology, MDD major depressive disorder, ROC receiver operating characteristic

The resulting single-feature model of IDS_30_ total score showed an excellent predictive performance when applied to the training set (AUC = 0.95), and a poor performance when extrapolated to the test set (AUC = 0.68) (Fig. 2). This suggests that while first-episode MDD patients could be accurately distinguished from subthreshold individuals who did not develop MDD based on the IDS_30_ total score alone, the differentiation was much more difficult between subthreshold individuals who developed and did not develop MDD as both groups had minimal symptoms resulting in more similar scores (Table 1).

### Analysis 2: model selection excluding IDS_30_ total score

To improve the model predictive performance on the extrapolation test set, we excluded IDS_30_ total score and repeated the analysis to allow other features to be selected (197 features). In this case, the number of features selected in a model ranged from 10 to 27, with a median of 14, demonstrating a degree of uncertainty in model selection (Fig. 1b). Twelve features were selected at least 90 times out of 100, among which nine features were selected 100 times. Sixteen features were selected at least once, and 169 features were never selected (Supplementary Table 3). We identified 17 unique models based on the combination of features selected (Supplementary Table 4). Two competing models consisting of 13 and 14 features occurred most frequently, 30 and 29 times, and had model probabilities of 0.22 and 0.50, respectively; the 13 features were a subset of the 14 features. This demonstrated that the frequency of occurrence did not necessarily correspond to the probability of being the best approximating model for the given data. The remaining unique models each occurred eight times or fewer. As there was considerable variability in feature selection and no strongly supported unique model, we implemented feature extraction and model averaging across all 100 models.

**Fig. 3:**
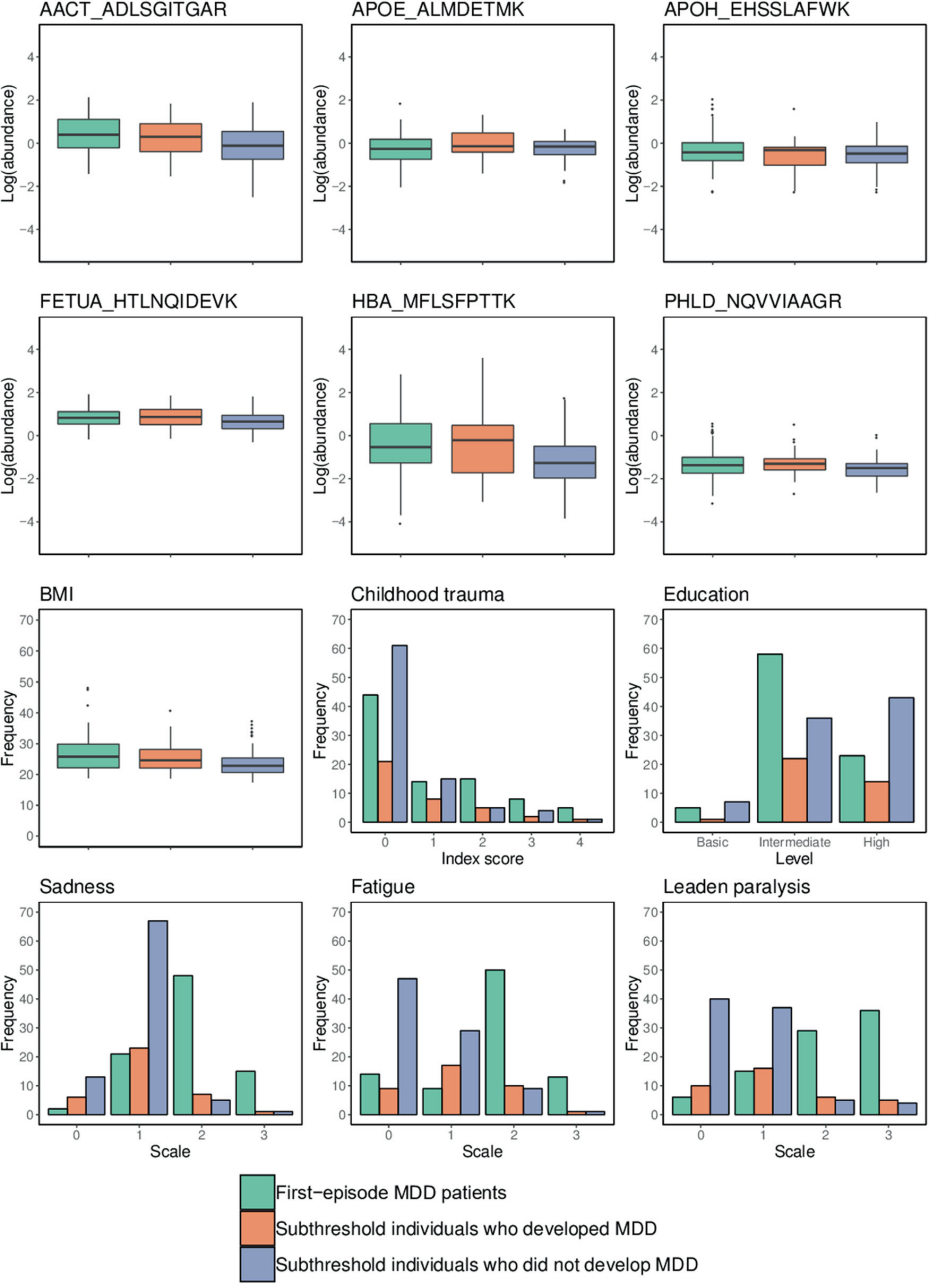
Disease indications of MDD represented by 12 features comprising Model 2. The distribution of data for individuals in the training set patient group (first-episode MDD patients), the extrapolation test set patient group (subthreshold symptomatic individuals who developed MDD within two or four years), and the shared reference group (subthreshold symptomatic individuals who did not develop MDD within four years) is shown. Protein abundances are represented by the log_2_-transformed peptide abundance ratios. The severity of depressive symptoms is represented on scale of 0 (none) to 3 (severe). Numeric features are illustrated using boxplots, and categorical features are illustrated using bar charts

The resulting average model (Model 2) was comprised of 12 features that had selection fractions greater than 0.9 (Table 2). Six peptides representing six proteins (alpha-1-antichymotrypsin (AACT), apolipoprotein E (APOE), apolipoprotein H (APOH), fetuin-A (FETUA), haemoglobin subunit alpha (HBA) and glycoprotein phospholipase D (PHLD)) were included, as well as three sociodemographic factors (BMI, childhood trauma and education level), and three depressive symptoms (sadness, fatigue and leaden paralysis). The 12-feature average prediction model showed an excellent predictive performance when applied to the training set (AUC = 0.94), and a fair predictive performance when extrapolated to the test set (AUC = 0.75) (Fig. 2). Here, the reduced performance on the latter can be explained by subthreshold individuals who developed MDD generally displaying weaker indications of disease (i.e., more similar to the reference group) compared to first-episode MDD patients (Fig. 3), as expected.

## Discussion

In this study, we evaluated the accuracy with which future onset of MDD could be predicted in subthreshold symptomatic individuals by extrapolating a disease prediction model of MDD that was trained to differentiate between first-episode MDD patients and subthreshold symptomatic individuals who did not develop MDD. We obtained a fair predictive performance (AUC = 0.75), which was promising given that it involved going beyond the scope of the model, although there is potential for improvement. Consequently, we demonstrated that some indicators of future MDD onset could be detected in subthreshold individuals using samples and data collected up to four years prior to diagnosis. This has important clinical implications with regards to enabling healthcare professionals to identify individuals with high probabilities of incident MDD and subsequently provide appropriate early intervention strategies, in light of accumulating evidence that subthreshold individuals have an increased risk of developing MDD^13,16–18^ and that subthreshold depression may represent a prodromal stage of MDD^43^.

The disease prediction model was comprised a combination of 12 proteomic, sociodemographic and clinical features. We identified six proteins (represented by six peptides) as biomarkers of MDD: AACT, APOE, APOH, FETUA, HBA and PHLD, which have functional roles in inflammatory response, lipid transport and metabolism, blood coagulation and oxygen transport^44^. Alterations in peripheral proteins involved in inflammatory response, the hypothalamic–pituitary–adrenal (HPA) axis, and carbohydrate and lipid metabolism have been reported in previous biomarker studies on depression^45–51^. Other apolipoproteins (apolipoprotein A, apolipoprotein B, apolipoprotein C-III and apolipoprotein D) have also been linked to depression^47,52–55^. It is worth mentioning that in this study, only the abundance levels of the peptides within these proteins and not necessarily those of the whole proteins were altered. In addition, it should be noted that some of the proteins identified in this study as biomarkers of MDD overlap with those identified in Cooper et al.^27^ as biomarkers of schizophrenia (APOE, APOH, FETUA and HBA). This is not entirely unexpected, not only because the same panel of protein peptides were used in both studies but also because different psychiatric disorders share common genetic predispositions^56^. Similarly, a review by Chan et al.^46^ has demonstrated that the same proteins have been identified as biomarkers of more than one major psychiatric disorder among MDD, schizophrenia and bipolar disorder.

We identified three sociodemographic factors (BMI, childhood trauma and education level) and three depressive symptoms (sadness, fatigue and leaden paralysis) as important predictors of MDD outcome. The relationship between BMI and depression is well known^57,58^, and some studies have reported a shared pathophysiology between obesity and depression, including dysregulation of the HPA axis and inflammatory response^59–61^. We found that MDD was associated with a higher childhood trauma index score, which measured experiences of emotional neglect, psychological abuse, physical abuse and sexual abuse in early life. Consistent with this, adverse or traumatic experience in childhood has been found as a strong risk factor for developing depression in adulthood^62–64^, and this relationship has been suggested to be reflected in disturbances in the neuroendocrine and autoimmune stress response system^65,66^. The link between education level and depression is less well established, with some studies reporting a decreased risk and others reporting an increased risk of depression with a higher education level^67–69^. Moreover, the identification of depressive symptoms as key predictors of MDD supports the idea that individual symptoms are associated with different risk factors, and that they are not interchangeable as assumed by the current diagnostic approach, in which symptoms are added together^70,71^. Note that sadness is one of the two core symptoms of depression according to both the DSM-5 and ICD-10, and was also required as a core symptom for the definition of subthreshold depression in this study. Fatigue (reduced energy level) is specified as a core symptom in the ICD-10, but not in the DSM-5. The identification of leaden paralysis (heaviness in arms and legs) as a key feature was interesting, as it is a symptom of atypical depression, a subtype of depression, and not included in the DSM-5 or ICD-10 criteria for general MDD. Overall, we demonstrated the advantage of integrating different aspects of patient data (i.e., proteomic, sociodemographic and clinical) for developing a clinically useful disease prediction model.

Furthermore, we demonstrated that the combined use of feature extraction and model averaging could effectively address model selection uncertainty and result in a parsimonious prediction model. In comparison with the one-feature model of IDS_30_ total score (Model 1) that was based on the dominant unique model in Analysis 1, the 12-feature model (Model 2) that was developed by implementing feature extraction and model averaging in Analysis 2 (in the absence of a dominant unique model) resulted in an improved predictive performance when applied to the extrapolation test set. Although the performance of the 12-feature model on the extrapolation test set was reduced compared with that on the training set, we considered the discrepancy to be largely due to the model having to go beyond its scope to make predictions on the test set, and less a result of model overfitting as we implemented several methods (LASSO regression, repeated tenfold cross-validation, feature extraction and model averaging) specifically to limit this. The utility of this method in producing reproducible predictions of a complex psychiatric disorder has been recently demonstrated by Cooper et al.^27^.

A limitation of this study is that models were trained on MDD patients given the limited availability of subthreshold individuals who developed MDD in the data set; we anticipate that model reproducibility would improve if training is conducted on the latter group. Small sample size is a major limitation in many psychiatric studies, due to the general difficulty associated with recruiting appropriate patient and reference samples. To ensure sufficient sample size for the present analysis, we employed a relatively liberal definition of subthreshold depression compared with other studies, allowing individuals experiencing mild and/or infrequent symptoms to be included as long as they fulfilled the specified criteria. In addition, we allowed a period of up to four years between initial assessment and subsequent diagnosis of MDD, but predictive performance may improve if a shorter period is examined. Finally, although we aimed to conduct a comprehensive analysis of the various features that could be associated with MDD outcome, other potentially important features may have been overlooked.

In conclusion, we investigated the prediction of future onset of depression in subthreshold symptomatic individuals using their proteomic, sociodemographic and clinical data. We developed a parsimonious 12-feature prediction model in the presence of model selection uncertainty by applying feature extraction and model averaging based on a set of candidate models. The results of this study suggest that early manifestations of depression, as represented by a combination of serum proteins, sociodemographic factors and depressive symptoms, can be detected in subthreshold individuals up to four years prior to clinical diagnosis. Having demonstrated that subthreshold individuals who developed MDD could be differentiated from those who did not develop MDD, further studies need to be conducted in subthreshold individuals for a better identification and characterisation of the condition to enable earlier interventions and improved outcomes.

## Supplementary information

Supplementary Information

## Data Availability

The R code that was used to generate the results can be made available upon request.
