# Supplementary material for: Integrating proteomic, sociodemographic and clinical data to predict future depression diagnosis in subthreshold symptomatic individuals
Source: Transl Psychiatry. 2019 Nov 7;9:277. doi: 10.1038/s41398-019-0623-2 (PMC6838310; doi:10.1038/s41398-019-0623-2)
Supplement: Supplementary file 1 — Supplementary Information [file 41398_2019_623_MOESM1_ESM.docx]

Supplementary Information

**Supplementary Methods**

**Clinical samples**

None of the participants selected for the present study were diagnosed with bipolar disorder, obsessive compulsive disorder, severe substance use disorder or psychotic disorder at the baseline assessment, and/or with bipolar disorder at the follow-up assessments. Comorbid anxiety disorder was not used as an exclusion criterion.

**Targeted protein quantification**

*Serum sample preparation*

Blood serum samples were stored at -80°C prior to analysis and prepared in a 96-well plate format using a Biomex NX liquid handler (Beckman Coulter, High Wycombe, UK). As described in Ozcan *et al.*(1), 5µL serum samples in each well were diluted with 105µL of 50mM ammonium bicarbonate. This was followed by disulphide bond reduction using 20.5µL of 32.5mM dithiothreitol (final concentration 5mM) and cysteine alkylation using 20.5µL of 75mM iodoacetamide (final concentration 10mM). Serum samples were then digested overnight using trypsin (1:50 for ratio of enzyme to protein weights). Stable isotope-labelled internal standard (SIS) peptides were spiked for each endogenous peptide.

Two hundred and nine serum samples of the selected participants were randomised to allocate equal numbers of reference and patient groups, training and test sets, and males and females across four plates.

*LC-MS analysis*

In this study, 146 peptides representing 77 proteins (Supplementary Table 2) were investigated using targeted multiple reaction monitoring (MRM) mass spectrometry (MS) analysis. Three to four interference-free transitions were selected for each peptide, as described previously(1).

Approximately 3.2µg of digested serum proteins was injected into an Agilent AdvanceBio Peptide Map column (2.1 × 150 mm 2.7-micron) and separated at 50^◦^C, as described previously(1). Peptides were eluted over a linear gradient from 3% to 30% acetonitrile in 0.1% formic acid in 45 minutes, at a flow rate of 0.3mL min^−1^. The mass spectra were acquired in positive mode and the quantification of all analytes was carried out in MRM mode(1). The researchers conducting the sample preparation and the MS analysis were completely blinded to the clinical status of the participants.

*Quality control samples*

We used quality control (QC) samples to assess technical variation associated with instrument performance and sample preparation. To assess the variation in instrument performance, a pooled QC sample was prepared by pooling together the digested clinical serum samples, and injected once a day along with the clinical samples for the duration of the entire study (28 injections in total). To asesss the variation in sample preparation, we used a commercial serum sample (Human Sera S7023, Sigma Aldrich); 24 Sigma serum QC samples were prepared following the same protocol as the clinical samples and distributed across the plates.

**Statistical analysis**

*MS data pre-processing*

In the processing of the raw mass spectrometry (MS) data using Skyline, peptide-transition peaks were examined manually, and peak integrations were adjusted accordingly where necessary, as described in Ozcan *et al.*(1). Peak area values of the endogenous and the SIS peptide-transitions were exported as a comma delimited data file for pre-processing and analysis using R. For each peptide, a quantifier transition was selected as the transition that was consistently most abundant in at least 80% of the sample runs in both the endogenous and SIS peptides. Peptides for which quantifier transitions could not be selected based on these criteria, which can occur in the presence of competing transitions, the raw peak data were examined for manual selection. Peptide quantification was based on the relative abundance of the endogenous and the SIS peptide-transitions, reported as the abundance ratio. This process, which is also referred to as ratio normalisation, was intended to account for any technical variation that occurred across MS runs. The abundance ratio was then log_2_-transformed for variance stabilisation.

*Statistical quality control*

We used principal component analysis (PCA) to identify any outlier samples based on the (log_2_-transformed) abundance ratios of 146 peptides (77 proteins) (Supplementary Figure 1). No outliers were identified. In addition, we used the geometric coefficient of variation (CV) to estimate the magnitude of variation in the proteomic data (in the original scale of measurement). For natural log-transformed data, the geometric CV was calculated as:

$$\mathrm{CV}=\sqrt{e^{{sd}^{2}-1}} \times100\%$$

where $sd$= standard deviation of the log-transformed data. To assess technical variation, we calculated the geometric CVs of peptide abundance across MS runs (within each plate) of pooled QC samples and Sigma serum QC samples. The median CV values were 6.7% and 22.9% for pooled QC and Sigma serum QC runs, respectively, based on the abundance ratios of 146 peptides. To assess biological variation, we calculated the geometric CVs of peptide abundance of clinical samples within each sample group. The median CV values were 59.2%, 51.7% and 49.7% for the training set patient group, extrapolation test set patient group and shared reference group, respectively.

*Multiple imputation*

We used multiple imputation to replace missing values. Methods used for imputation were: predictive mean matching for numeric data, logistic regression imputation for binary data (factor with two levels), and multinomial logistic regression imputation for categorical data (factor with more than two levels). The final complete dataset was produced by finding the central estimate of the missing values across five imputed datasets, using the median and the mode for numeric and categorical variables, respectively.

*Akaike information criterion and Akaike weights*

The general formula for the $\mathrm{AIC}$(2,3) is:

$$\mathrm{AIC}= -2L+2k$$

where $L$ = log-likelihood, $k$ = number of features selected in a model.

In the present study, we used the bias-corrected version of $\mathrm{AIC}$, the $\mathrm{AIC}_{c}$(4,5):

$$\mathrm{AIC}_{c}=\mathrm{AIC}+ \frac{2k\left( k+1 \right)}{n-k-1}$$

where *n* = sample size, $k$ = number of features selected in a model.

For a given model $i$ in a set of $R$ models, the Akaike weight, $w_{i}$, was computed using the difference between its $\mathrm{AIC}_{c}$ and the lowest $\mathrm{AIC}_{c}$(3,6):

$$\Delta_{i}=\mathrm{AIC}_{c i}- \mathrm{AIC}_{c min}$$

$$w_{i}= \frac{exp(-\frac{1}{2}\Delta_{i})}{\sum_{r=1}^{R} exp(-\frac{1}{2}\Delta_{r})}$$

Note that the weight was a value between 0 and 1, and the sum of weights of all models was equal to 1.

**Supplementary Tables & Figures**

**Supplementary Table 1. Nine DSM-5 depressive symptoms and corresponding IDS_30_ items.** For a diagnosis of MDD under the DSM-5, five or more of the nine symptoms need to be present, including at least one core symptom (*) of depressed mood or anhedonia, during the same two-week period and represent a change from previous functioning(7). The present study defined subthreshold symptomatic individuals at baseline using 16 items of the IDS_30_ ^(ref)^(8) that correspond to the nine DSM symptom domains (and comprise the shortened version of the IDS_30_, the 16-item QIDS(9)): individuals had to present two or more depressive symptoms including one core symptom, whereby any one of the corresponding items had to be above zero for a symptom to be considered as present. Abbreviations: DSM (Diagnostic and Statistical Manual of Mental Disorders); IDS (Inventory of Depressive Symptomatology); MDD (major depressive disorder); QIDS (Quick Inventory of Depressive Symptomatology).

| *DSM-5 symptom* | *IDS item* | *IDS description* |
| --- | --- | --- |
| *Depressed mood | IDS 5 | Sadness |
| *Anhedonia | IDS 19 | General interest |
| Decrease or increase in weight or appetite | IDS 11  IDS 12  IDS 13  IDS 14 | Decreased appetite  Increased appetite  Decreased weight  Increased weight |
| Insomnia or hypersomnia | IDS 1  IDS 2  IDS 3  IDS 4 | Early insomnia  Middle insomnia  Late insomnia  Hypersomnia |
| Psychomotor agitation or retardation | IDS 23  IDS 24 | Psychomotor retardation  Psychomotor agitation |
| Fatigue or loss of energy | IDS 20 | Energy level |
| Worthlessness or inappropriate guilt | IDS 16 | Self-criticism |
| Diminished ability to concentrate or indecisiveness | IDS 15 | Concentration/decision-making |
| Recurrent thoughts of death or suicide | IDS 18 | Thoughts of death or suicide |

**Supplementary Table 2. A summary of the 77 proteins (146 peptides) investigated.** The UniProt(10) accession number and the protein ID are shown for each peptide.

| *UniProt accession number* | *Protein* | *Peptide* |
| --- | --- | --- |
| P01009 | A1AT | SVLGQLGITK |
| P01009 | A1AT | SPLFMGK |
| P01009 | A1AT | LSITGTYDLK |
| P04217 | A1BG | ATWSGAVLAGR |
| P04217 | A1BG | SGLSTGWTQLSK |
| P04217 | A1BG | CLAPLEGAR |
| P08697 | A2AP | DFLQSLK |
| P08697 | A2AP | DSFHLDEQFTVPVEMMQAR |
| P08697 | A2AP | FDPSLTQR |
| P01023 | A2MG | NEDSLVFVQTDK |
| P01023 | A2MG | AIGYLNTGYQR |
| P01011 | AACT | ADLSGITGAR |
| P01011 | AACT | EQLSLLDR |
| P01011 | AACT | EIGELYLPK |
| P02768 | ALBU | AAFTECCQAADK |
| P02768 | ALBU | QNCELFEQLGEYK |
| P02768 | ALBU | ETYGEMADCCAK |
| P02760 | AMBP | ETLLQDFR |
| P02760 | AMBP | TVAACNLPIVR |
| P01019 | ANGT | ALQDQLVLVAAK |
| P01019 | ANGT | SLDFTELDVAAEK |
| P01019 | ANGT | FMQAVTGWK |
| P01008 | ANT3 | FDTISEK |
| P01008 | ANT3 | LPGIVAEGR |
| P02647 | APOA1 | ATEHLSTLSEK |
| P02647 | APOA1 | EQLGPVTQEFWDNLEK |
| P02652 | APOA2 | SPELQAEAK |
| P06727 | APOA4 | ISASAEELR |
| P06727 | APOA4 | ALVQQMEQLR |
| P06727 | APOA4 | IDQNVEELK |
| P02654 | APOC1 | EFGNTLEDK |
| P02654 | APOC1 | EWFSETFQK |
| P02655 | APOC2 | TAAQNLYEK |
| P02655 | APOC2 | ESLSSYWESAK |
| P02656 | APOC3 | DALSSVQESQVAQQAR |
| P02656 | APOC3 | GWVTDGFSSLK |
| P55056 | APOC4 | AWFLESK |
| P05090 | APOD | VLNQELR |
| P02649 | APOE | LEEQAQQIR |
| P02649 | APOE | SELEEQLTPVAEETR |
| P02649 | APOE | LGPLVEQGR |
| P02649 | APOE | AATVGSLAGQPLQER |
| P02649 | APOE | ALMDETMK |
| Q13790 | APOF | SLPTEDCENEK |
| P02749 | APOH | VSFFCK |
| P02749 | APOH | EHSSLAFWK |
| O14791 | APOL1 | VNEPSILEMSR |
| O14791 | APOL1 | VTEPISAESGEQVER |
| O14791 | APOL1 | LNILNNNYK |
| O95445 | APOM | AFLLTPR |
| O95445 | APOM | SLTSCLDSK |
| P02747 | C1QC | TNQVNSGGVLLR |
| P00736 | C1R | YTTEIIK |
| P09871 | C1S | LLEVPEGR |
| P09871 | C1S | TNFDNDIALVR |
| P04003 | C4BPA | YTCLPGYVR |
| P04003 | C4BPA | EDVYVVGTVLR |
| P04003 | C4BPA | FSAICQGDGTWSPR |
| P00915 | CAH1 | ADGLAVIGVLMK |
| P08185 | CBG | ITQDAQLK |
| P08185 | CBG | GTWTQPFDLASTR |
| Q96IY4 | CBPB2 | YPLYVLK |
| Q96IY4 | CBPB2 | DTGTYGFLLPER |
| O43866 | CD5L | EATLQDCPSGPWGK |
| P00450 | CERU | EVGPTNADPVCLAK |
| P00450 | CERU | NNEGTYYSPNYNPQSR |
| P00751 | CFAB | YGLVTYATYPK |
| P00751 | CFAB | DLLYIGK |
| P00751 | CFAB | DISEVVTPR |
| P00751 | CFAB | EELLPAQDIK |
| P08603 | CFAH | CFEGFGIDGPAIAK |
| P10909 | CLUS | IDSLLENDR |
| P10909 | CLUS | FMETVAEK |
| P06681 | CO2 | HAIILLTDGK |
| P01024 | CO3 | TGLQEVEVK |
| P01024 | CO3 | AGDFLEANYMNLQR |
| P01024 | CO3 | VYAYYNLEESCTR |
| P0C0L4 | CO4A | VLSLAQEQVGGSPEK |
| P0C0L4 | CO4A | DFALLSLQVPLK |
| P0C0L4 | CO4A | ITQVLHFTK |
| P0C0L5 | CO4B | VGDTLNLNLR |
| P13671 | CO6 | TLNICEVGTIR |
| P13671 | CO6 | SEYGAALAWEK |
| P07357 | CO8A | MESLGITSR |
| P02748 | CO9 | VVEESELAR |
| P02748 | CO9 | LSPIYNLVPVK |
| P00748 | FA12 | CFEPQLLR |
| P00748 | FA12 | VVGGLVALR |
| O75636 | FCN3 | YGIDWASGR |
| P02765 | FETUA | HTLNQIDEVK |
| P02765 | FETUA | FSVVYAK |
| P02751 | FINC | YSFCTDHTVLVQTR |
| P06396 | GELS | AGALNSNDAFVLK |
| P06396 | GELS | SEDCFILDHGK |
| P69905 | HBA | FLASVSTVLTSK |
| P69905 | HBA | MFLSFPTTK |
| P02790 | HEMO | VDGALCMEK |
| P02790 | HEMO | NFPSPVDAAFR |
| P05546 | HEP2 | IAIDLFK |
| P05546 | HEP2 | FAFNLYR |
| P00738 | HPT | VTSIQDWVQK |
| P00738 | HPT | DYAEVGR |
| P00738 | HPT | VGYVSGWGR |
| P04196 | HRG | DSPVLIDFFEDTER |
| P04196 | HRG | ADLFYDVEALDLESPK |
| P05155 | IC1 | FQPTLLTLPR |
| P01876 | IGHA1 | TPLTATLSK |
| P01876 | IGHA1 | DASGVTFTWTPSSGK |
| P01877 | IGHA2 | DASGATFTWTPSSGK |
| P01857 | IGHG1 | FNWYVDGVEVHNAK |
| P01859 | IGHG2 | GLPAPIEK |
| P01860 | IGHG3 | NQVSLTCLVK |
| P01860 | IGHG3 | DTLMISR |
| P01871 | IGHM | YAATSQVLLPSK |
| P01871 | IGHM | QIQVSWLR |
| P19827 | ITIH1 | LDAQASFLPK |
| P19827 | ITIH1 | GSLVQASEANLQAAQDFVR |
| P19823 | ITIH2 | IQPSGGTNINEALLR |
| P19823 | ITIH2 | FYNQVSTPLLR |
| Q14624 | ITIH4 | GPDVLTATVSGK |
| Q14624 | ITIH4 | ETLFSVMPGLK |
| P03952 | KLKB1 | LSMDGSPTR |
| P01042 | KNG1 | DIPTNSPELEETLTHTITK |
| P51884 | LUM | SLEDLQLTHNK |
| P36955 | PEDF | DTDTGALLFIGK |
| P36955 | PEDF | TVQAVLTVPK |
| P36955 | PEDF | ELLDTVTAPQK |
| P36955 | PEDF | LQSLFDSPDFSK |
| Q96PD5 | PGRP2 | TFTLLDPK |
| P80108 | PHLD | NQVVIAAGR |
| P00747 | PLMN | FVTWIEGVMR |
| P02753 | RET4 | QEELCLAR |
| P02743 | SAMP | IVLGQEQDSYGGK |
| P04278 | SHBG | IALGGLLFPASNLR |
| P04278 | SHBG | LPLVPALDGCLR |
| P05452 | TETN | EQQALQTVCLK |
| P00734 | THRB | SGIECQLWR |
| P00734 | THRB | ELLESYIDGR |
| P02787 | TRFE | EGYYGYTGAFR |
| P02766 | TTHY | AADDTWEPFASGK |
| P02766 | TTHY | VLDAVR |
| P02774 | VTDB | VLEPTLK |
| P02774 | VTDB | HLSLLTTLSNR |
| P02774 | VTDB | THLPEVFLSK |
| P04004 | VTNC | DWHGVPGQVDAAMAGR |
| P04004 | VTNC | DVWGIEGPIDAAFTR |

**Supplementary Table 3.** **A summary of 198 features analysed for model selection.** The selection fractions of 146 proteomic, 22 sociodemographic and 30 clinical features in Analysis 1 (model selection including IDS_30_ total score) and Analysis 2 (model selection excluding IDS_30_ total score) are shown. The features included in the prediction models in each analysis (Model 1 and Model 2; Table 2) are shown in bold. Proteomic features are represented in a Protein_Peptide format.

| *Feature* | *Selection fraction* | |
| --- | --- | --- |
|  | *Analysis 1* | *Analysis 2* |
| ***Proteomic*** | | |
| A1AT_LSITGTYDLK | 0.00 | 0.00 |
| A1AT_SPLFMGK | 0.00 | 0.00 |
| A1AT_SVLGQLGITK | 0.00 | 0.00 |
| A1BG_ATWSGAVLAGR | 0.00 | 0.00 |
| A1BG_CLAPLEGAR | 0.00 | 0.00 |
| A1BG_SGLSTGWTQLSK | 0.00 | 0.00 |
| A2AP_DFLQSLK | 0.00 | 0.00 |
| A2AP_DSFHLDEQFTVPVEMMQAR | 0.00 | 0.00 |
| A2AP_FDPSLTQR | 0.00 | 0.00 |
| A2MG_AIGYLNTGYQR | 0.00 | 0.00 |
| A2MG_NEDSLVFVQTDK | 0.00 | 0.63 |
| **AACT_ADLSGITGAR** | 0.00 | **1.00** |
| AACT_EIGELYLPK | 0.00 | 0.77 |
| AACT_EQLSLLDR | 0.00 | 0.00 |
| ALBU_AAFTECCQAADK | 0.00 | 0.00 |
| ALBU_ETYGEMADCCAK | 0.00 | 0.00 |
| ALBU_QNCELFEQLGEYK | 0.00 | 0.00 |
| AMBP_ETLLQDFR | 0.00 | 0.00 |
| AMBP_TVAACNLPIVR | 0.00 | 0.00 |
| ANGT_ALQDQLVLVAAK | 0.00 | 0.00 |
| ANGT_FMQAVTGWK | 0.00 | 0.00 |
| ANGT_SLDFTELDVAAEK | 0.00 | 0.00 |
| ANT3_FDTISEK | 0.00 | 0.00 |
| ANT3_LPGIVAEGR | 0.00 | 0.00 |
| APOA1_ATEHLSTLSEK | 0.00 | 0.00 |
| APOA1_EQLGPVTQEFWDNLEK | 0.00 | 0.00 |
| APOA2_SPELQAEAK | 0.00 | 0.00 |
| APOA4_ALVQQMEQLR | 0.00 | 0.00 |
| APOA4_IDQNVEELK | 0.00 | 0.00 |
| APOA4_ISASAEELR | 0.00 | 0.00 |
| APOC1_EFGNTLEDK | 0.00 | 0.00 |
| APOC1_EWFSETFQK | 0.00 | 0.00 |
| APOC2_ESLSSYWESAK | 0.00 | 0.00 |
| APOC2_TAAQNLYEK | 0.00 | 0.00 |
| APOC3_DALSSVQESQVAQQAR | 0.00 | 0.00 |
| APOC3_GWVTDGFSSLK | 0.00 | 0.00 |
| APOC4_AWFLESK | 0.00 | 0.00 |
| APOD_VLNQELR | 0.00 | 0.00 |
| APOE_AATVGSLAGQPLQER | 0.00 | 0.00 |
| **APOE_ALMDETMK** | 0.00 | **0.99** |
| APOE_LEEQAQQIR | 0.00 | 0.00 |
| APOE_LGPLVEQGR | 0.00 | 0.00 |
| APOE_SELEEQLTPVAEETR | 0.00 | 0.00 |
| APOF_SLPTEDCENEK | 0.00 | 0.00 |
| **APOH_EHSSLAFWK** | 0.00 | **1.00** |
| APOH_VSFFCK | 0.00 | 0.00 |
| APOL1_LNILNNNYK | 0.00 | 0.00 |
| APOL1_VNEPSILEMSR | 0.00 | 0.12 |
| APOL1_VTEPISAESGEQVER | 0.00 | 0.00 |
| APOM_AFLLTPR | 0.00 | 0.00 |
| APOM_SLTSCLDSK | 0.00 | 0.00 |
| C1QC_TNQVNSGGVLLR | 0.00 | 0.00 |
| C1R_YTTEIIK | 0.00 | 0.00 |
| C1S_LLEVPEGR | 0.00 | 0.00 |
| C1S_TNFDNDIALVR | 0.00 | 0.00 |
| C4BPA_EDVYVVGTVLR | 0.00 | 0.00 |
| C4BPA_FSAICQGDGTWSPR | 0.00 | 0.00 |
| C4BPA_YTCLPGYVR | 0.00 | 0.00 |
| CAH1_ADGLAVIGVLMK | 0.00 | 0.00 |
| CBG_GTWTQPFDLASTR | 0.00 | 0.00 |
| CBG_ITQDAQLK | 0.00 | 0.00 |
| CBPB2_DTGTYGFLLPER | 0.00 | 0.00 |
| CBPB2_YPLYVLK | 0.00 | 0.00 |
| CD5L_EATLQDCPSGPWGK | 0.00 | 0.00 |
| CERU_EVGPTNADPVCLAK | 0.00 | 0.00 |
| CERU_NNEGTYYSPNYNPQSR | 0.00 | 0.00 |
| CFAB_DISEVVTPR | 0.00 | 0.00 |
| CFAB_DLLYIGK | 0.00 | 0.00 |
| CFAB_EELLPAQDIK | 0.00 | 0.00 |
| CFAB_YGLVTYATYPK | 0.00 | 0.00 |
| CFAH_CFEGFGIDGPAIAK | 0.00 | 0.00 |
| CLUS_FMETVAEK | 0.00 | 0.00 |
| CLUS_IDSLLENDR | 0.00 | 0.00 |
| CO2_HAIILLTDGK | 0.00 | 0.00 |
| CO3_AGDFLEANYMNLQR | 0.00 | 0.00 |
| CO3_TGLQEVEVK | 0.00 | 0.00 |
| CO3_VYAYYNLEESCTR | 0.00 | 0.00 |
| CO4A_DFALLSLQVPLK | 0.00 | 0.00 |
| CO4A_ITQVLHFTK | 0.00 | 0.00 |
| CO4A_VLSLAQEQVGGSPEK | 0.00 | 0.00 |
| CO4B_VGDTLNLNLR | 0.00 | 0.00 |
| CO6_SEYGAALAWEK | 0.00 | 0.08 |
| CO6_TLNICEVGTIR | 0.00 | 0.00 |
| CO8A_MESLGITSR | 0.00 | 0.21 |
| CO9_LSPIYNLVPVK | 0.00 | 0.00 |
| CO9_VVEESELAR | 0.00 | 0.00 |
| FA12_CFEPQLLR | 0.00 | 0.00 |
| FA12_VVGGLVALR | 0.00 | 0.00 |
| FCN3_YGIDWASGR | 0.00 | 0.02 |
| FETUA_FSVVYAK | 0.00 | 0.00 |
| **FETUA_HTLNQIDEVK** | 0.01 | **0.97** |
| FINC_YSFCTDHTVLVQTR | 0.00 | 0.00 |
| GELS_AGALNSNDAFVLK | 0.00 | 0.00 |
| GELS_SEDCFILDHGK | 0.00 | 0.00 |
| HBA_FLASVSTVLTSK | 0.00 | 0.34 |
| **HBA_MFLSFPTTK** | 0.00 | **1.00** |
| HEMO_NFPSPVDAAFR | 0.00 | 0.00 |
| HEMO_VDGALCMEK | 0.00 | 0.00 |
| HEP2_FAFNLYR | 0.00 | 0.00 |
| HEP2_IAIDLFK | 0.00 | 0.00 |
| HPT_DYAEVGR | 0.00 | 0.00 |
| HPT_VGYVSGWGR | 0.00 | 0.00 |
| HPT_VTSIQDWVQK | 0.00 | 0.00 |
| HRG_ADLFYDVEALDLESPK | 0.00 | 0.21 |
| HRG_DSPVLIDFFEDTER | 0.00 | 0.00 |
| IC1_FQPTLLTLPR | 0.00 | 0.00 |
| IGHA1_DASGVTFTWTPSSGK | 0.00 | 0.00 |
| IGHA1_TPLTATLSK | 0.00 | 0.00 |
| IGHA2_DASGATFTWTPSSGK | 0.00 | 0.00 |
| IGHG1_FNWYVDGVEVHNAK | 0.00 | 0.00 |
| IGHG2_GLPAPIEK | 0.01 | 0.26 |
| IGHG3_DTLMISR | 0.00 | 0.00 |
| IGHG3_NQVSLTCLVK | 0.00 | 0.00 |
| IGHM_QIQVSWLR | 0.00 | 0.00 |
| IGHM_YAATSQVLLPSK | 0.00 | 0.00 |
| ITIH1_GSLVQASEANLQAAQDFVR | 0.00 | 0.00 |
| ITIH1_LDAQASFLPK | 0.00 | 0.00 |
| ITIH2_FYNQVSTPLLR | 0.00 | 0.00 |
| ITIH2_IQPSGGTNINEALLR | 0.00 | 0.00 |
| ITIH4_ETLFSVMPGLK | 0.00 | 0.00 |
| ITIH4_GPDVLTATVSGK | 0.00 | 0.18 |
| KLKB1_LSMDGSPTR | 0.00 | 0.00 |
| KNG1_DIPTNSPELEETLTHTITK | 0.00 | 0.00 |
| LUM_SLEDLQLTHNK | 0.00 | 0.00 |
| PEDF_DTDTGALLFIGK | 0.00 | 0.00 |
| PEDF_ELLDTVTAPQK | 0.00 | 0.00 |
| PEDF_LQSLFDSPDFSK | 0.00 | 0.00 |
| PEDF_TVQAVLTVPK | 0.00 | 0.00 |
| PGRP2_TFTLLDPK | 0.00 | 0.00 |
| **PHLD_NQVVIAAGR** | 0.04 | **1.00** |
| PLMN_FVTWIEGVMR | 0.00 | 0.00 |
| RET4_QEELCLAR | 0.01 | 0.00 |
| SAMP_IVLGQEQDSYGGK | 0.00 | 0.00 |
| SHBG_IALGGLLFPASNLR | 0.00 | 0.00 |
| SHBG_LPLVPALDGCLR | 0.01 | 0.00 |
| TETN_EQQALQTVCLK | 0.00 | 0.00 |
| THRB_ELLESYIDGR | 0.00 | 0.00 |
| THRB_SGIECQLWR | 0.00 | 0.00 |
| TRFE_EGYYGYTGAFR | 0.00 | 0.00 |
| TTHY_AADDTWEPFASGK | 0.00 | 0.00 |
| TTHY_VLDAVR | 0.00 | 0.00 |
| VTDB_HLSLLTTLSNR | 0.00 | 0.00 |
| VTDB_THLPEVFLSK | 0.00 | 0.00 |
| VTDB_VLEPTLK | 0.00 | 0.00 |
| VTNC_DVWGIEGPIDAAFTR | 0.00 | 0.00 |
| VTNC_DWHGVPGQVDAAMAGR | 0.00 | 0.00 |
|  | | |
| ***Sociodemographic*** | | |
| Sex | 0.00 | 0.00 |
| Age | 0.00 | 0.00 |
| **BMI** | 0.00 | **1.00** |
| **Education** | 0.00 | **0.93** |
| Physical activity | 0.00 | 0.00 |
| Smoking | 0.00 | 0.00 |
| Alcohol abuse | 0.00 | 0.00 |
| Weekly alcohol consumption | 0.00 | 0.00 |
| Recreational drug use (past month) | 0.00 | 0.00 |
| Partner | 0.00 | 0.00 |
| Children | 0.00 | 0.00 |
| Employment | 0.00 | 0.00 |
| Absent from work due to health problems (past 6 months) | 0.00 | 0.00 |
| Childhood life event index score | 0.00 | 0.11 |
| **Childhood trauma index score** | 0.00 | **1.00** |
| Number of negative life events (past year) | 0.00 | 0.00 |
| Family history | 0.00 | 0.00 |
| Heart disease | 0.00 | 0.00 |
| Diabetes | 0.00 | 0.00 |
| Other chronic disease | 0.00 | 0.00 |
| Anti-inflammatory drug | 0.00 | 0.00 |
| Heart medication | 0.00 | 0.00 |
|  |  |  |
| ***Clinical*** | | |
| Early insomnia | 0.00 | 0.00 |
| Midle insomnia | 0.00 | 0.00 |
| Late insomnia | 0.00 | 0.00 |
| Hypersomnia | 0.00 | 0.00 |
| **Sadness** | 0.00 | **1.00** |
| Irritability | 0.00 | 0.00 |
| Anxiety | 0.00 | 0.00 |
| Mood reactivity | 0.00 | 0.19 |
| Diurnal mood variation | 0.00 | 0.00 |
| Mood quality | 0.00 | 0.07 |
| Increased weight/appetite | 0.00 | 0.00 |
| Decreased weight/appetite | 0.00 | 0.00 |
| Concentration/decision-making | 0.00 | 0.00 |
| Self-criticism | 0.00 | 0.26 |
| Pessimism | 0.00 | 0.00 |
| Suicidal thoughts | 0.00 | 0.02 |
| General interest | 0.00 | 0.00 |
| **Fatigue** | 0.00 | **1.00** |
| Pleasure/enjoyment | 0.00 | 0.15 |
| Sexual interest | 0.00 | 0.00 |
| Psychomotor retardation | 0.00 | 0.00 |
| Psychomotor agitation | 0.00 | 0.00 |
| Somatic pains | 0.00 | 0.00 |
| Other bodily symptoms | 0.00 | 0.00 |
| Panic/phobic symptoms | 0.00 | 0.00 |
| Gastrointestinal problems | 0.00 | 0.00 |
| Interpersonal sensitivity | 0.00 | 0.00 |
| **Leaden paralysis** | 0.00 | **1.00** |
| IDS_30_ severity classification | 0.00 | 0.00 |
| **IDS_30_ total score** | **1.00** | - |

**Supplementary Table 4. Unique models based on the combinations of features selected.** Three unique models were identified in Analysis 1 (model selection including IDS_30_ total score), and 17 unique models were identified in Analysis 2 (model selection excluding IDS_30_ total score).

| *Unique model* | *Number of features* | *Frequency* | *Model probability* |
| --- | --- | --- | --- |
| ***Analysis 1*** | | | |
| IDS_30_ total score | 1 | 96 | 0.9825 |
| PHLD_NQVVIAAGR + IDS_30_ total score | 2 | 3 | 0.0171 |
| FETUA_HTLNQIDEVK + IGHG2_GLPAPIEK + PHLD_NQVVIAAGR + RET4_QEELCLAR + SHBG_LPLVPALDGCLR + IDS_30_ total score | 6 | 1 | 0.0004 |
|  | | | |
| ***Analysis 2*** | | | |
| AACT_ADLSGITGAR + AACT_EIGELYLPK + APOE_ALMDETMK + APOH_EHSSLAFWK + FETUA_HTLNQIDEVK + HBA_MFLSFPTTK + PHLD_NQVVIAAGR + Education + BMI + Childhood trauma + Sadness + Fatigue + Leaden paralysis | 13 | 30 | 0.2169 |
| A2MG_NEDSLVFVQTDK + AACT_ADLSGITGAR + AACT_EIGELYLPK + APOE_ALMDETMK + APOH_EHSSLAFWK + FETUA_HTLNQIDEVK + HBA_MFLSFPTTK + PHLD_NQVVIAAGR + Education + BMI + Childhood trauma + Sadness + Fatigue + Leaden paralysis | 14 | 29 | 0.5024 |
| A2MG_NEDSLVFVQTDK + AACT_ADLSGITGAR + APOE_ALMDETMK + APOH_EHSSLAFWK + APOL1_VNEPSILEMSR + CO8A_MESLGITSR + FETUA_HTLNQIDEVK + HBA_FLASVSTVLTSK + HBA_MFLSFPTTK + HRG_ADLFYDVEALDLESPK + IGHG2_GLPAPIEK + ITIH4_GPDVLTATVSGK + PHLD_NQVVIAAGR + Education + BMI + Childhood life event + Childhood trauma + Sadness + Mood reactivity + Self-criticism + Fatigue + Pleasure/enjoyment + Leaden paralysis | 23 | 3 | 4.08E-10 |
| A2MG_NEDSLVFVQTDK + AACT_ADLSGITGAR + APOE_ALMDETMK + APOH_EHSSLAFWK + APOL1_VNEPSILEMSR + CO6_SEYGAALAWEK + CO8A_MESLGITSR + FETUA_HTLNQIDEVK + HBA_FLASVSTVLTSK + HBA_MFLSFPTTK + HRG_ADLFYDVEALDLESPK + IGHG2_GLPAPIEK + ITIH4_GPDVLTATVSGK + PHLD_NQVVIAAGR + Education + BMI + Childhood life event + Childhood trauma + Sadness + Mood reactivity + Mood quality + Self-criticism + Fatigue + Pleasure/enjoyment + Leaden paralysis | 25 | 5 | 1.29E-11 |
| A2MG_NEDSLVFVQTDK + AACT_ADLSGITGAR + AACT_EIGELYLPK + APOE_ALMDETMK + APOH_EHSSLAFWK + FETUA_HTLNQIDEVK + HBA_FLASVSTVLTSK + HBA_MFLSFPTTK + PHLD_NQVVIAAGR + Education + BMI + Childhood trauma + Sadness + Fatigue + Leaden paralysis | 15 | 8 | 0.1530 |
| AACT_ADLSGITGAR + AACT_EIGELYLPK + APOE_ALMDETMK + APOH_EHSSLAFWK + HBA_MFLSFPTTK + PHLD_NQVVIAAGR + BMI + Childhood trauma + Sadness + Fatigue + Leaden paralysis | 11 | 2 | 0.0158 |
| AACT_ADLSGITGAR + AACT_EIGELYLPK + APOE_ALMDETMK + APOH_EHSSLAFWK + FETUA_HTLNQIDEVK + HBA_MFLSFPTTK + PHLD_NQVVIAAGR + BMI + Childhood trauma + Sadness + Fatigue + Leaden paralysis | 12 | 4 | 0.0963 |
| A2MG_NEDSLVFVQTDK + AACT_ADLSGITGAR + AACT_EIGELYLPK + APOE_ALMDETMK + APOH_EHSSLAFWK + FETUA_HTLNQIDEVK + HBA_FLASVSTVLTSK + HBA_MFLSFPTTK + IGHG2_GLPAPIEK + PHLD_NQVVIAAGR + Education + BMI + Childhood trauma + Sadness + Self-criticism + Fatigue + Leaden paralysis | 17 | 3 | 0.0002 |
| A2MG_NEDSLVFVQTDK + AACT_ADLSGITGAR + APOE_ALMDETMK + APOH_EHSSLAFWK + CO8A_MESLGITSR + FETUA_HTLNQIDEVK + HBA_FLASVSTVLTSK + HBA_MFLSFPTTK + HRG_ADLFYDVEALDLESPK + IGHG2_GLPAPIEK + ITIH4_GPDVLTATVSGK + PHLD_NQVVIAAGR + Education + BMI + Childhood trauma + Sadness + Mood reactivity + Self-criticism + Fatigue + Pleasure/enjoyment + Leaden paralysis | 21 | 3 | 1.03E-08 |
| A2MG_NEDSLVFVQTDK + AACT_ADLSGITGAR + APOE_ALMDETMK + APOH_EHSSLAFWK + CO8A_MESLGITSR + FETUA_HTLNQIDEVK + HBA_FLASVSTVLTSK + HBA_MFLSFPTTK + HRG_ADLFYDVEALDLESPK + IGHG2_GLPAPIEK + PHLD_NQVVIAAGR + Education + BMI + Childhood trauma + Sadness + Self-criticism + Fatigue + Leaden paralysis | 18 | 2 | 3.67E-05 |
| A2MG_NEDSLVFVQTDK + AACT_ADLSGITGAR + APOE_ALMDETMK + APOH_EHSSLAFWK + APOL1_VNEPSILEMSR + CO6_SEYGAALAWEK + CO8A_MESLGITSR + FCN3_YGIDWASGR + FETUA_HTLNQIDEVK + HBA_FLASVSTVLTSK + HBA_MFLSFPTTK + HRG_ADLFYDVEALDLESPK + IGHG2_GLPAPIEK + ITIH4_GPDVLTATVSGK + PHLD_NQVVIAAGR + Education + BMI + Childhood life event + Childhood trauma + Sadness + Mood reactivity + Mood quality + Self-criticism + Suicidal thoughts + Fatigue + Pleasure/enjoyment + Leaden paralysis | 27 | 2 | 2.44E-14 |
| A2MG_NEDSLVFVQTDK + AACT_ADLSGITGAR + APOE_ALMDETMK + APOH_EHSSLAFWK + APOL1_VNEPSILEMSR + CO6_SEYGAALAWEK + CO8A_MESLGITSR + FETUA_HTLNQIDEVK + HBA_FLASVSTVLTSK + HBA_MFLSFPTTK + HRG_ADLFYDVEALDLESPK + IGHG2_GLPAPIEK + ITIH4_GPDVLTATVSGK + PHLD_NQVVIAAGR + Education + BMI + Childhood life event + Childhood trauma + Sadness + Mood reactivity + Self-criticism + Fatigue + Pleasure/enjoyment + Leaden paralysis | 24 | 1 | 2.61E-11 |
| A2MG_NEDSLVFVQTDK + AACT_ADLSGITGAR + APOE_ALMDETMK + APOH_EHSSLAFWK + FETUA_HTLNQIDEVK + HBA_FLASVSTVLTSK + HBA_MFLSFPTTK + IGHG2_GLPAPIEK + PHLD_NQVVIAAGR + Education + BMI + Childhood trauma + Sadness + Self-criticism + Fatigue + Leaden paralysis | 16 | 2 | 0.0006 |
| AACT_ADLSGITGAR + AACT_EIGELYLPK + APOH_EHSSLAFWK + HBA_MFLSFPTTK + PHLD_NQVVIAAGR + BMI + Childhood trauma + Sadness + Fatigue + Leaden paralysis | 10 | 1 | 0.0148 |
| A2MG_NEDSLVFVQTDK + AACT_ADLSGITGAR + APOE_ALMDETMK + APOH_EHSSLAFWK + CO8A_MESLGITSR + FETUA_HTLNQIDEVK + HBA_FLASVSTVLTSK + HBA_MFLSFPTTK + HRG_ADLFYDVEALDLESPK + IGHG2_GLPAPIEK + ITIH4_GPDVLTATVSGK + PHLD_NQVVIAAGR + Education + BMI + Childhood trauma + Sadness + Mood reactivity + Self-criticism + Fatigue + Leaden paralysis | 20 | 3 | 7.79E-07 |
| A2MG_NEDSLVFVQTDK + AACT_ADLSGITGAR + APOE_ALMDETMK + APOH_EHSSLAFWK + APOL1_VNEPSILEMSR + CO8A_MESLGITSR + FETUA_HTLNQIDEVK + HBA_FLASVSTVLTSK + HBA_MFLSFPTTK + HRG_ADLFYDVEALDLESPK + IGHG2_GLPAPIEK + ITIH4_GPDVLTATVSGK + PHLD_NQVVIAAGR + Education + BMI + Childhood trauma + Sadness + Mood reactivity + Self-criticism + Fatigue + Pleasure/enjoyment + Leaden paralysis | 22 | 1 | 6.91E-10 |
| A2MG_NEDSLVFVQTDK + AACT_ADLSGITGAR + APOE_ALMDETMK + APOH_EHSSLAFWK + CO8A_MESLGITSR + FETUA_HTLNQIDEVK + HBA_FLASVSTVLTSK + HBA_MFLSFPTTK + HRG_ADLFYDVEALDLESPK + IGHG2_GLPAPIEK + PHLD_NQVVIAAGR + Education + BMI + Childhood trauma + Sadness + Mood reactivity + Self-criticism + Fatigue + Leaden paralysis | 19 | 1 | 2.24E-07 |

**Supplementary Figure 1. Principal component analysis (PCA) plot of protein peptide abundance ratios of clinical samples.** The first two principal components are plotted with the percentage of variation accounted for by each principal component shown in the axis labels. Data points are coloured according to the allocation of clinical samples into the training set patient group (first-episode MDD patients), the extrapolation test set patient group (subthreshold symptomatic individuals who developed MDD within two or four years) and the shared reference group (subthreshold symptomatic individuals who did not develop MDD within four years). Abbreviations: MDD (major depressive disorder); PC (principal component).

**References**

1. Ozcan S, et al. Towards reproducible MRM based biomarker discovery using dried blood spots. Sci Rep. 2017 Mar 27;7:45178.

2. Akaike H. Information theory and an extension of the maximum likelihood principle. In: Petrov BN, Csaki F, editors. Second International Symposium on Information Theory. Budapest: Akadémiai Kiado; 1973. p. 267–81.

3. Burnham KP, Anderson DR. Model Selection and Multi-Model Inference: A Practical Information-Theoretical Approach. 2nd ed. Springer; 2002. 496 p.

4. Hurvich CM, Tsai CL. Regression and time series model selection in small samples. Biometrika. 1989 Jun 1;76(2):297–307.

5. Sugiura N. Further analysis of the data by akaike’ s information criterion and the finite corrections. Commun Stat - Theory Methods. 1978 Jan 27;7(1):13–26.

6. Burnham KP, Anderson DR. Multimodel Inference: Understanding AIC and BIC in Model Selection. Sociol Methods Res. 2004;33(2):261–304.

7. American Psychiatric Association. Diagnostic and Statistical Manual of Mental Disorders, fifth edition. American Psychiatric Association; 2013.

8. Rush AJ, Gullion CM, Basco MR, Jarrett RB, Trivedi MH. The Inventory of Depressive Symptomatology (IDS): psychometric properties. Psychol Med. 1996 May;26(03):477.

9. Rush AJ, et al. The 16-item Quick Inventory of Depressive Symptomatology (QIDS), clinician rating (QIDS-C), and self-report (QIDS-SR): A psychometric evaluation in patients with chronic major depression. Biol Psychiatry. 2003;54(5):573–83.

10. The UniProt Consortium. UniProt: a hub for protein information. Nucleic Acids Res. 2015;
